# Frequency of Complicated Appendicitis and Associated Risk Factors Among Patients With Acute Appendicitis in Taiz, Yemen

**DOI:** 10.7759/cureus.113106

**Published:** 2026-07-21

**Authors:** Sina A Al-Nomi, Omar Mohammed Abdullatif Saif, Shaheed A Alaamry, Hamdah ALomeri, Samer Al Bothaigi

**Affiliations:** 1 General Surgery, Faculty of Medicine and Health Sciences, Taiz University, Taiz, YEM; 2 General Surgery, Al-Jamhouri Teaching Hospital, Taiz, YEM

**Keywords:** acute appendicitis, appendicolith, complicated appendicitis, delayed presentation, emergency surgery, misdiagnosis, yemen

## Abstract

Background

Complicated appendicitis - defined here as gangrenous appendicitis, perforation, peri-appendicular abscess, or generalized purulent peritonitis - causes substantial surgical morbidity where access to emergency care is constrained. This study determined the frequency and associated factors among patients with acute appendicitis in Taiz, Yemen.

Methods

This hospital-based, prospective, two-center, observational analytical study was conducted at the Authority of Al-Thawra Hospital and Al-Jumhoori Teaching Hospital in Taiz, Yemen, from February 2024 to February 2025. Consecutive patients of any age who underwent appendectomy for intraoperatively confirmed acute appendicitis were enrolled. Complication status was classified from macroscopic intraoperative findings. Associations were evaluated using bivariate analysis and multivariable binary logistic regression.

Results

A total of 207 patients were included, of whom 49 (23.7%) had complicated appendicitis. Pre-hospital delay showed a strong crude association with complicated appendicitis (crude odds ratio (cOR): 26.57, 95% confidence interval (CI): 9.04-78.08), whereas in-hospital delay was not statistically significant. Pre-hospital delay was not entered into the multivariable model because its major component causes were analyzed separately. In multivariable analysis, misdiagnosis at the primary healthcare level (adjusted odds ratio (aOR): 19.98, 95% CI: 7.56-52.80), consultation with a traditional healer (aOR: 22.11, 95% CI: 4.54-107.63), poor recognition of symptom severity (aOR: 18.08, 95% CI: 4.63-70.59), comorbidity (aOR: 7.13, 95% CI: 1.81-28.06), and appendicolith (aOR: 3.95, 95% CI: 1.24-12.58) were independently associated with complicated appendicitis. The apparent area under the receiver operating characteristic curve (AUC) was 0.879, the optimism-corrected value was 0.860, the Hosmer-Lemeshow p-value was 0.994, and the Brier score was 0.108.

Conclusions

Among 207 patients, 49 (23.7%) had complicated appendicitis. The strongest independent associations involved prior primary-level misdiagnosis, traditional-healer consultation, poor recognition of symptom severity, comorbidity, and appendicolith. These observational findings identify potentially modifiable points in the care pathway but do not establish causality. They support the evaluation of earlier recognition and referral strategies in conflict-affected Yemen.

## Introduction

Acute appendicitis is one of the most common indications for emergency abdominal surgery and remains an important contributor to the global burden of time-sensitive surgical disease [1-3]. Its clinical course is not uniform. Some patients present with localized uncomplicated inflammation, whereas others progress to gangrene, perforation, peri-appendicular abscess, or generalized peritonitis [4-7]. These advanced presentations, commonly grouped as complicated appendicitis, are associated with greater operative difficulty, infectious morbidity, antimicrobial exposure, hospital use, and cost [2,6-8].

The distinction between uncomplicated and complicated appendicitis is therefore practical rather than merely descriptive. It influences the urgency of intervention, the need for source control, the duration of antimicrobial therapy, and the intensity of postoperative monitoring [6-8]. Current World Society of Emergency Surgery guidance emphasizes risk stratification because selected uncomplicated cases may be suitable for non-operative treatment, whereas complicated appendicitis generally requires urgent operative or procedural source control [7].

Time from symptom onset to definitive surgical care is a central determinant of disease severity. Prospective and observational studies show that rupture becomes more likely when symptoms remain untreated, particularly beyond the first 24-48 hours [9,10]. However, delay is not a single clinical event. A short supervised interval after hospital assessment, resuscitation, and antibiotics is biologically different from untreated delay in the community or at a non-definitive first-contact facility [11-13].

In low-resource settings, pre-hospital delay is rarely explained by patient choice alone. It may reflect poverty, distance, transport barriers, limited health literacy, fear of surgery, reliance on non-biomedical care, restricted diagnostic capacity at first-contact facilities, and fragmented referral systems [14-19]. Misdiagnosis at the primary healthcare level is especially important because the patient has already entered the formal health system, but the opportunity for early recognition and referral has been missed [15,17].

Yemen’s prolonged conflict has damaged health infrastructure, disrupted supply chains, reduced workforce availability, intensified household poverty, and constrained access to emergency surgical care [20,21]. Taiz’s public referral hospitals serve patients from urban neighborhoods and rural districts within this strained system. Conflict-related disruption may extend decision, first-contact, referral, and transport intervals before definitive surgery. The present study did not measure every conflict-related barrier directly; this context provides the rationale for examining prospectively recorded care-pathway variables.

To our knowledge, this is the first prospective two-center analytical study in Taiz to estimate the frequency of complicated appendicitis while separately examining composite pre-hospital delay, specific reported reasons for delay, and adjusted associations with operative severity. The primary objective was to determine the frequency of complicated appendicitis among patients undergoing appendectomy at two public referral hospitals. The secondary objective was to identify demographic, clinical, delay-related, and intraoperative factors associated with complicated appendicitis.

## Materials and methods

Study design and setting

This was a hospital-based, prospective, two-center, observational analytical study conducted at the Authority of Al-Thawra Hospital and Al-Jumhoori Teaching Hospital in Taiz city, Yemen (approval no. ECTU2024/20-6). These institutions are major public referral centers for emergency surgery in the governorate and receive patients from urban and rural catchment areas.

A prospective design was used because symptom timing, care-seeking pathways, and reasons for pre-hospital delay are often incompletely recorded in routine notes and are more reliably captured at the time of care.

Study period and participants

Participant recruitment and data collection were undertaken from February 2024 to February 2025. Patients of either sex and any age were eligible if they underwent appendectomy and had macroscopic intraoperative evidence of acute appendicitis.

Patients were excluded if they were treated non-operatively, if surgery demonstrated an alternative diagnosis, if the appendix appeared normal and appendicitis was not supported clinically or histologically where available, or if consent for participation was not obtained.

Sampling strategy, enrollment flow, and sample size

A consecutive non-probability sampling strategy was applied. All patients undergoing appendectomy for suspected acute appendicitis during the study period were screened for eligibility. Patients were included when acute appendicitis was confirmed macroscopically at operation and consent was obtained. Patients managed non-operatively, patients with an alternative operative diagnosis, patients with a normal-appearing appendix without clinical or histological support for appendicitis where available, and patients without consent were excluded. The final analytic cohort comprised 207 eligible patients with complete primary analysis variables.

A separate screening log of all non-enrolled appendectomy candidates and their specific exclusion counts was not maintained. For that reason, an exact CONSORT-style flow diagram could not be constructed; this is now acknowledged as a reporting limitation. Sample size planning was based on estimating the proportion of complicated appendicitis among surgically confirmed cases using a single-proportion formula.

Although this sample was adequate for estimating the primary frequency outcome, the number of complicated cases was modest relative to the six predictors in the final logistic regression model. The magnitude of adjusted associations should therefore be interpreted cautiously and externally validated in larger cohorts.

Variables and operational definitions

The dependent variable was complicated appendicitis. Complicated appendicitis was defined by at least one of the following macroscopic intraoperative findings: gangrenous appendicitis, perforated appendicitis, peri-appendicular abscess, or generalized purulent peritonitis. Uncomplicated appendicitis comprised catarrhal or phlegmonous inflammation without gangrene, perforation, abscess, or generalized purulent peritonitis. This operational framework was aligned with published pathological descriptions, World Society of Emergency Surgery guidance, and the American Association for the Surgery of Trauma emergency general surgery appendicitis severity framework [4-7].

Independent variables included age, sex, residence area, comorbidity, any delay, pre-hospital delay, in-hospital delay, reported causes of pre-hospital delay, and appendicolith. Age was recorded in completed years. Residence was classified as urban or rural according to the patient-reported place of residence. Comorbidity was defined as any previously diagnosed chronic medical condition documented by history or available records at presentation, including hypertension, diabetes mellitus, chronic kidney disease, malignancy, or another chronic condition judged clinically relevant. Because individual comorbidity categories were sparse, comorbidity was analyzed as a binary variable indicating any comorbidity versus none.

Any delay indicated pre-hospital delay, in-hospital delay, or both. Pre-hospital delay was defined as more than 24 hours from symptom onset to arrival at the study hospital for definitive surgical evaluation, consistent with thresholds used in appendicitis literature [14,22,23]. In-hospital delay was recorded prospectively as a binary study-form variable for clinically meaningful delay between hospital admission and appendectomy; the form did not retain a continuous hour-based interval.

Misdiagnosis at the primary healthcare level was defined as seeking formal healthcare before reaching the study hospital and receiving an alternative diagnosis or management plan that did not identify suspected acute appendicitis. Consultation with a traditional healer was defined as seeking advice or treatment from a traditional or non-biomedical practitioner before hospital presentation. Poor recognition of symptom severity was defined as failure by the patient or family to recognize persistent or worsening symptoms as requiring urgent medical evaluation. Other specific causes of pre-hospital delay referred to reported delay causes not included in those three main categories, including difficulty reaching the hospital or transport barriers, financial barriers, fear of surgery, and other less frequent patient-reported causes. These were grouped descriptively because the individual subgroups were small. Appendicolith was defined as a firm intraluminal concretion identified intraoperatively [24,25].

Diagnostic confirmation and severity classification

The primary diagnosis of acute appendicitis and the complicated-versus-uncomplicated severity classification were established from macroscopic intraoperative findings recorded immediately after appendectomy by the operating surgical team. Preoperative ultrasonography or computed tomography, when obtained during routine care, was not used as the definitive study outcome. Histopathology, where available, could support the diagnosis of appendicitis but was not required for the operative severity classification. Consequently, the study endpoint was an intraoperative clinical classification rather than a uniform histopathological endpoint.

To improve standardization, the same predefined severity categories were used at both hospitals throughout data collection. Operative findings were documented by the operating surgeon or responsible surgical team immediately after appendectomy. When the macroscopic category was uncertain, the classification was reviewed with a senior member of the surgical team using the study definitions before data entry. The study did not use a central blinded adjudication panel, and this has been added to the limitations.

Data collection

Data were collected prospectively using a structured case record form developed for the study. The case record form captured demographic information, baseline clinical characteristics, comorbidity, symptom timing, sequence of care-seeking contacts, hospital arrival, pre-hospital and in-hospital delay variables, reported reasons for pre-hospital delay, and intraoperative findings. The form was not reproduced in the article because of space limitations, but its domains are described here to support reproducibility, and the form can be made available by the corresponding author if requested by the journal.

Information on symptom onset and care-seeking pathway was obtained directly from the patient whenever feasible and from an accompanying relative when the patient was a child, severely unwell, or otherwise unable to provide a reliable history. Operative findings were documented immediately after appendectomy.

Data quality assurance

The data collection form was used in a standardized manner across both hospitals. Eligibility was based on operative confirmation rather than admission diagnosis, reducing outcome misclassification. Delay-related information was collected prospectively rather than reconstructed solely from case notes. Completed forms were reviewed for completeness and internal consistency before data entry. No missing data were identified for the primary analysis variables.

Statistical analysis

Data were entered into Microsoft Excel (Microsoft® Corp., Redmond, WA, USA) and analyzed using IBM SPSS Statistics for Windows, Version 26 (Released 2018; IBM Corp., Armonk, NY, USA) and R software (R Foundation for Statistical Computing, Vienna, Austria). Categorical variables were summarized as frequencies and percentages. Continuous variables were assessed for normality using the Shapiro-Wilk test. Because age was not normally distributed, it was reported as the median and interquartile range.

Associations between categorical predictors and complicated appendicitis were assessed using the Pearson chi-square test or Fisher's exact test when expected cell counts were small. Age was compared using the Mann-Whitney U test. Crude odds ratios (cORs) with 95% confidence intervals (CIs) were calculated.

Variables considered clinically relevant and/or statistically associated in bivariate analysis were considered for multivariable binary logistic regression. Adjusted odds ratios (aORs) with 95% CIs were reported. The final model was specified as a component-level pathway model and included residence, appendicolith, comorbidity, misdiagnosis at the primary healthcare level, consultation with a traditional healer, and poor recognition of symptom severity. The composite pre-hospital delay variable was not entered into this main model because it overlapped conceptually with its component causes and because the number of complicated cases was limited. Therefore, the adjusted component associations should not be interpreted as proving effects independent of total symptom-to-hospital time. The absence of a separate adjusted sensitivity model replacing the component causes with composite pre-hospital delay is now treated as a major analytic limitation.

Model discrimination was assessed using the area under the receiver operating characteristic curve (AUC). Calibration was assessed using the Hosmer-Lemeshow goodness-of-fit test and the Brier score. Collinearity was assessed using variance inflation factor (VIF) and tolerance statistics. A two-sided p-value < 0.05 was considered statistically significant. Internal validation was performed using 1,000 bootstrap samples to estimate the optimism-corrected AUC.

## Results

Participant characteristics

A total of 207 surgically managed patients with intraoperatively confirmed acute appendicitis were included. Of these, 134 of 207 patients (64.7%) were managed at the Authority of Al-Thawra Hospital and 73 of 207 patients (35.3%) at Al-Jumhoori Teaching Hospital.

The cohort included 108 of 207 males (52.2%) and 99 of 207 females (47.8%). The median age was 20 years (interquartile range: 15-27 years). Most patients were urban residents, with 143 of 207 patients (69.1%), whereas 64 of 207 patients (30.9%) were rural residents. Chronic comorbidity was recorded in 16 of 207 patients (7.7%). Baseline and delay-related characteristics are summarized in Table 1.

**Table 1:** Baseline and delay-related characteristics of the study cohort (N = 207) Note: Percentages are based on the total cohort unless otherwise stated. A single hyphen indicates not applicable. IQR: interquartile range

Characteristic	Category/statistic	n or value	% or IQR
Sex	Male	108	52.2
	Female	99	47.8
Age	Median years	20	-
	IQR years	15-27	-
Residence	Urban	143	69.1
	Rural	64	30.9
Any comorbidity	Yes	16	7.7
Any delay	Yes	96	46.4
Pre-hospital delay	Yes	92	44.4
In-hospital delay	Yes	11	5.3
Misdiagnosis at primary healthcare level	Yes	45	21.7
Poor recognition of symptom severity	Yes	14	6.8
Traditional healer consultation	Yes	9	4.3
Other specific causes of pre-hospital delay	Yes	24	11.6

Frequency and operative severity of appendicitis

Complicated appendicitis was identified in 49 of 207 patients (23.7%). The remaining 158 of 207 patients (76.3%) had uncomplicated appendicitis. Uncomplicated disease included catarrhal and phlegmonous cases. Complicated presentations included diffuse peritonitis, gangrene, and appendicular abscess. Appendicolith-related appendicitis was identified in 23 of 207 patients (11.1%).

Bivariate associations with complicated appendicitis

Selected bivariate associations are presented in Table 2. Complicated appendicitis occurred in 30 of 108 males (27.8%) and 19 of 99 females (19.2%), with no statistically significant association between sex and complicated disease (cOR: 1.62, 95% CI: 0.84-3.11, p = 0.147). Median age was 22 years in the complicated group and 20 years in the uncomplicated group; this difference was not statistically significant (p = 0.305).

**Table 2:** Selected bivariate associations with complicated appendicitis Note: Reference categories were female sex, urban residence, no comorbidity, no appendicolith, no delay, no primary misdiagnosis, no traditional-healer consultation, and no poor recognition of symptom severity, as applicable. Fisher's exact test was used when expected cell counts were small. A single hyphen indicates not applicable. *Statistically significant p-value (p < 0.05). CA: complicated appendicitis; UA: uncomplicated appendicitis; cOR: crude odds ratio; CI: confidence interval

Predictor	Category	Total, n	CA, n	CA, %	UA, n	UA %	cOR	CI lower	CI upper	p-value
Sex	Male	108	30	27.8	78	72.2	1.62	0.84	3.11	0.147
Sex	Female	99	19	19.2	80	80.8	Reference	-	-	0.147
Residence	Rural	64	22	34.4	42	65.6	2.25	1.16	4.37	0.015*
Any comorbidity	Yes	16	10	62.5	6	37.5	6.25	2.22	19.00	< 0.001*
Appendicolith	Yes	23	12	52.2	11	47.8	4.33	1.77	10.60	0.001*
Any delay	Yes	96	45	46.9	51	53.1	23.60	8.05	69.20	< 0.001*
Pre-hospital delay	Yes	92	45	48.9	47	51.1	26.57	9.04	78.08	< 0.001*
In-hospital delay	Yes	11	5	45.5	6	54.5	2.88	0.84	9.88	0.136
Primary misdiagnosis	Yes	45	27	60.0	18	40.0	9.55	4.52	20.10	< 0.001*
Traditional healer	Yes	9	5	55.6	4	44.4	4.38	1.13	17.00	0.036*
Poor recognition	Yes	14	8	57.1	6	42.9	4.94	1.62	15.00	0.005*

Rural residence was associated with complicated appendicitis in a crude analysis. Complicated disease occurred in 22 of 64 rural residents (34.4%) compared with 27 of 143 urban residents (18.9%; cOR: 2.25, 95% CI: 1.16-4.37, p = 0.015). Comorbidity was also associated with complicated appendicitis: 10 of 16 patients with comorbidity (62.5%) had complicated disease compared with 39 of 191 patients without comorbidity (20.4%; cOR: 6.25, 95% CI: 2.22-19.00, p < 0.001).

Delay-related variables showed the strongest crude associations. Any delay was present in 45 of 49 patients with complicated appendicitis (91.8%) compared with 51 of 158 patients with uncomplicated appendicitis (32.3%; cOR: 23.60, 95% CI: 8.05-69.20, p < 0.001). Pre-hospital delay was present in 45 of 49 patients with complicated appendicitis (91.8%) compared with 47 of 158 patients with uncomplicated appendicitis (29.7%; cOR: 26.57, 95% CI: 9.04-78.08, p < 0.001). In-hospital delay was present in 5 of 49 patients with complicated appendicitis (10.2%) and 6 of 158 patients with uncomplicated appendicitis (3.8%), and the association was not statistically significant (cOR: 2.88, 95% CI: 0.84-9.88, p = 0.136).

Multivariable logistic regression

Variables entered into the final multivariable model were residence, appendicolith, comorbidity, misdiagnosis at the primary healthcare level, consultation with a traditional healer, and poor recognition of symptom severity. Pre-hospital delay was excluded because its main component causes were included separately. Crude and adjusted estimates for all six predictors are presented side by side in Table 3.

**Table 3:** Multivariable logistic regression analysis of factors associated with complicated appendicitis (N = 207) Note: Reference categories were urban residence, no appendicolith, no comorbidity, no primary-level misdiagnosis, no traditional-healer consultation, and no poor recognition of symptom severity. The p-values are from the adjusted model. *Statistically significant p-value (p < 0.05). cOR: crude odds ratio; aOR: adjusted odds ratio; CI: confidence interval

Predictor	cOR	cOR CI lower	cOR CI upper	aOR	aOR CI lower	aOR CI upper	p-value
Rural residence	2.25	1.16	4.37	2.01	0.83	4.89	0.125
Appendicolith	4.33	1.77	10.60	3.95	1.24	12.58	0.020*
Comorbidity	6.25	2.22	19.00	7.13	1.81	28.06	0.005*
Primary misdiagnosis	9.55	4.52	20.10	19.98	7.56	52.80	< 0.001*
Traditional healer	4.38	1.13	17.00	22.11	4.54	107.63	< 0.001*
Poor recognition	4.94	1.62	15.00	18.08	4.63	70.59	< 0.001*

After adjustment, primary-level misdiagnosis (aOR: 19.98, 95% CI: 7.56-52.80, p < 0.001), consultation with a traditional healer (aOR: 22.11, 95% CI: 4.54-107.63, p < 0.001), poor recognition of symptom severity (aOR: 18.08, 95% CI: 4.63-70.59, p < 0.001), comorbidity (aOR: 7.13, 95% CI: 1.81-28.06, p = 0.005), and appendicolith (aOR: 3.95, 95% CI: 1.24-12.58, p = 0.020) remained independently associated with complicated appendicitis. Rural residence did not retain statistical significance (aOR: 2.01, 95% CI: 0.83-4.89, p = 0.125).

Model performance

The final multivariable logistic regression model showed good apparent internal performance (Table 4). The overall likelihood-ratio test was statistically significant (chi-square = 83.42, df = 6, p < 0.001). The apparent AUC was 0.879, and the bias-corrected AUC after 1,000 bootstrap samples was 0.860. The Hosmer-Lemeshow test did not suggest lack of fit (chi-square = 1.41, df = 8, p = 0.994). The Brier score was 0.108, and VIF values ranged from 1.04 to 1.34.

**Table 4:** Apparent internal performance of the final multivariable model Note: These statistics represent apparent/internal performance and do not substitute for external validation. *Statistically significant p-value (p < 0.05). AUC: area under the receiver operating characteristic curve; VIF: variance inflation factor

Metric	Statistic	Value
Overall likelihood-ratio model test	Chi-square	83.42
	df	6
	p-value	< 0.001*
McFadden pseudo-R^2^	Coefficient	0.368
Apparent AUC	AUC	0.879
Bias-corrected AUC	AUC	0.860
Hosmer-Lemeshow test	Chi-square	1.410
	df	8
	p-value	0.994
Brier score	Score	0.108
VIF range	Range	1.04-1.34

## Discussion

Principal findings

This prospective, two-center study found that complicated appendicitis accounted for 49 of 207 surgically confirmed cases (23.7%). The adjusted model identified primary-level misdiagnosis, traditional-healer consultation, poor recognition of symptom severity, comorbidity, and appendicolith as independently associated factors. Pre-hospital delay had a strong crude association, whereas in-hospital delay was not statistically significant.

The findings indicate that operative severity in this cohort reflected both disease biology and the pathway to definitive care. The wide confidence intervals for several pathway variables, particularly traditional-healer consultation and poor recognition of symptom severity, show that the direction of association was strong but the precise magnitude remains uncertain.

Comparison with other settings and study contribution

The observed frequency of 23.7% closely approximates the pooled 23% reported for lower-middle-income countries and is lower than the pooled 34% reported for low-income countries in a systematic review of 87 studies including 25,582 participants [26]. In a Swiss multicenter cohort, complicated appendicitis was reported in 20% of 241 pre-pandemic patients and 52% of 65 patients during the lockdown period [27]. These comparisons support the plausibility of the Taiz estimate but should be interpreted cautiously because definitions, age distributions, imaging access, referral patterns, and operative thresholds differ between studies.

The present study adds context-specific evidence by prospectively separating pre-hospital from in-hospital delay, identifying specific reported causes of pre-hospital delay, and presenting crude and adjusted associations with an intraoperatively defined outcome. To our knowledge, this combination has not previously been reported in a prospective, two-center analytical study from Taiz.

Pre-hospital delay and the local care pathway

Pre-hospital delay was the strongest crude pathway variable. Patients with pre-hospital delay had markedly higher odds of complicated disease, whereas in-hospital delay was not statistically significant. A short supervised interval after hospital arrival, with assessment, resuscitation, antibiotics, and operative planning, is different from untreated time in the community or repeated non-definitive contacts [11-13,22,23].

In Yemen, damaged infrastructure, constrained transport and fuel availability, household out-of-pocket costs, and fragmented referral systems are plausible mechanisms through which conflict may prolong access to definitive surgery [20,21]. The present dataset directly measured some pathway factors, including misdiagnosis, traditional-healer consultation, and poor recognition, but did not measure exact travel time, road closures, household income, or self-medication. Those unmeasured factors should therefore be regarded as contextual hypotheses rather than demonstrated mediators in this cohort.

Primary-level misdiagnosis

Misdiagnosis at the primary healthcare level was one of the strongest independent associations. These patients had sought biomedical care, but suspected appendicitis was not recognized or escalated. Early appendicitis can resemble gastroenteritis, urinary tract infection, gynecological disease, or nonspecific abdominal pain, particularly when laboratory and imaging support are limited [15-17]. In comparable resource-constrained settings, delayed presentation and diagnostic error have also been linked to more advanced disease [14,16]. Simple referral-oriented criteria and explicit return precautions for persistent or worsening abdominal pain may therefore deserve evaluation.

Traditional-healer consultation and symptom recognition

Traditional-healer consultation and poor recognition of symptom severity were independently associated with complicated appendicitis. These results should not be reduced to individual blame. Non-biomedical providers may be geographically closer, less costly, or more trusted, and families may initially interpret abdominal pain as a self-limiting illness. However, symptom-directed remedies cannot reverse fixed luminal obstruction or established transmural inflammation. Community messaging and collaboration with trusted local providers could be evaluated as referral interventions, but the present observational study cannot establish their effectiveness.

Health-literacy interventions should be concrete. Persistent or worsening abdominal pain, migration to the right lower abdomen, fever, vomiting, or inability to tolerate oral intake should prompt urgent evaluation. Such recommendations are consistent with the observed association between poor recognition and advanced disease, while acknowledging that access barriers may remain even after danger symptoms are recognized [18,19].

Comorbidity, appendicolith, and rural residence

Comorbidity remained independently associated with complicated appendicitis, although only 16 of 207 patients (7.7%) had a recorded chronic condition. Chronic illness may alter symptom interpretation, reduce physiological reserve, or complicate triage. The small subgroup and wide confidence interval require cautious interpretation.

Appendicolith was independently associated with complicated disease, consistent with evidence that a fixed obstructive lesion is associated with more severe appendicitis and a higher likelihood of appendectomy after initial antibiotic treatment [24,25]. When imaging identifies an appendicolith, prolonged observation should therefore be approached cautiously.

Rural residence was associated with complicated appendicitis in crude analysis but not after adjustment. This attenuation is compatible with a mediated pathway in which geography operates through recognition, first-contact diagnosis, referral, and transport rather than acting as an independent biological determinant.

Strengths and limitations

Strengths include prospective data collection, enrolment at two public referral hospitals, consecutive sampling, immediate operative classification, complete primary analysis variables, presentation of both crude and adjusted estimates, and bootstrap assessment of model optimism.

Several limitations remain. The study was hospital-based and included only patients who reached the two hospitals and underwent appendectomy; it therefore does not represent all appendicitis in Taiz and is vulnerable to referral and selection bias. A formal screening log of all non-enrolled appendectomy candidates was not retained, so the exact enrollment flow and exclusion counts cannot be reconstructed. Symptom timing and reasons for delay relied on patient or family recall. The study did not use a uniform preoperative imaging protocol, a uniform histopathological endpoint, or a central blinded adjudication panel for operative severity. The binary in-hospital-delay variable did not retain an exact hour threshold.

A major analytic limitation is that the final multivariable model excluded the composite pre-hospital delay variable and did not include a separate adjusted sensitivity model replacing the component delay causes with total pre-hospital delay. This choice reduced conceptual overlap between the composite exposure and its component causes, but it weakens any attempt to separate the influence of specific pathway factors from total elapsed untreated time. Accordingly, misdiagnosis, traditional-healer consultation, and poor symptom recognition should be interpreted as component-level pathway markers associated with complicated disease, not as variables proven to act independently of total symptom-to-hospital time.

Several exposure groups were small, producing wide confidence intervals, and 49 outcome events were available for six model predictors. Educational level, household income, exact travel time, road access, number of prior healthcare contacts, self-medication, pre-referral antibiotics, and postoperative outcomes were not measured. Finally, the model was developed and internally assessed in the same dataset; it is not a validated clinical decision rule. Because the design was observational, all reported relationships are associations and should not be interpreted as proof of causation.

Future research

Larger multicenter studies across Yemen should confirm the frequency of complicated appendicitis and externally validate the adjusted associations. Future data collection should separate decision delay, first-contact delay, referral delay, and transport delay; record imaging and histopathology systematically; and include postoperative outcomes, length of stay, readmission, mortality, and household cost. Mixed-methods work could clarify how trust, cost, insecurity, self-medication, and referral practices shape the care pathway.

## Conclusions

Among 207 patients undergoing appendectomy in Taiz, 49 (23.7%) had complicated appendicitis. Pre-hospital delay showed a strong crude association with complicated appendicitis, whereas in-hospital delay was not statistically significant. In the component-level adjusted model, primary-level misdiagnosis, traditional-healer consultation, poor recognition of symptom severity, comorbidity, and appendicolith were associated with complicated disease.

These findings identify care pathways and clinical markers associated with operative severity but do not establish causality. Because composite pre-hospital delay was not included in a separate adjusted sensitivity model, the pathway variables should be interpreted as markers within the delayed care pathway rather than as factors proven to act independently of total elapsed symptom-to-hospital time. The results support the evaluation of interventions that improve early symptom recognition, first-contact assessment, and referral to definitive surgical care. Larger external cohorts are required before the model is used for prediction or policy decisions.
